# Empowering Patient Safety: Assessment of Adverse Drug Reaction Knowledge and Practice Among Pharmacy Professionals

**DOI:** 10.3390/pharmacy13010001

**Published:** 2024-12-29

**Authors:** Josipa Bukić, Dario Leskur, Toni Durdov, Joško Božić, Darko Modun, Ana Šešelja Perišin, Daniela Ančić, Martina Šepetavc, Ante Mihanović, Doris Rušić

**Affiliations:** 1 Department of Pharmacy, School of Medicine, University of Split, 21000 Split, Croatia; jbukic@mefst.hr (J.B.); dleskur@mefst.hr (D.L.); toni.durdov@mefst.hr (T.D.); jbozic@mefst.hr (J.B.); dmodun@mefst.hr (D.M.); aperisin@mefst.hr (A.Š.P.); daniela.vukasovic@gmail.com (D.A.); amihanov@mefst.hr (A.M.); drusic@mefst.hr (D.R.); 2 Department of Laboratory Medicine and Pharmacy, Faculty of Medicine, Josip Juraj Strossmayer University of Osijek, 31000 Osijek, Croatia; 3 Department of Pathophysiology, School of Medicine, University of Split, 21000 Split, Croatia; jbozic@mefst.hr; 4 Farmacia Pharmacy,10000 Zagreb, Croatia; martina.sepetavc@atlanticgrupa.com; 5 Split-Dalmatia County Pharmacy, 21000 Split, Croatia

**Keywords:** adverse drug reaction, drug safety, pharmacists, pharmacovigilance

## Abstract

Despite technological advancements, healthcare professionals must actively prioritize patient safety. Reporting adverse drug reactions is a critical aspect of this responsibility, and the most accessible healthcare providers, community pharmacists, and pharmacy technicians play a key role. Therefore, this study assessed their knowledge and practices regarding adverse drug reaction reporting in Croatia. A total of 180 participants were included. Pharmacists demonstrated significantly better knowledge than technicians (94.78 vs. 73.97, *p* = 0.024). Chronic medication users also showed greater understanding compared to non-users (104.96 vs. 85.39, *p* = 0.021). Knowledge improved with the number of adverse drug reactions reported, and most participants (72.78%) had reported adverse drug reactions. Pharmacists were 83.60% more likely to report adverse drug reactions than technicians (*p* < 0.001). These findings reveal a gap in pharmacy technicians’ integration into pharmacovigilance, underscoring a need to strengthen their role in adverse drug reaction reporting and patient safety.

## 1. Introduction

The initial data on drug efficacy and safety, which originate from randomized controlled clinical trials, do not provide insight into the real-world situation once the drug is on the market [1]. Clinical trials involved in the studying of medicines include a relatively small number of selected individuals for a short period of time. There are adverse drug reactions which may only emerge once the medicine is used by heterogeneous population and over a long period of time [2]. The extensive pharmacovigilance legislation set the guidance for the establishment and maintenance of quality assured pharmacovigilance systems for marketing authorization holders and regulatory authorities throughout good pharmacovigilance practices (GVP) modules [3]. These systems can effectively monitor the safety of authorized medicinal products and detect any change to their risk–benefit balance if they gather safety data from all relevant sources. Drug safety monitoring and data from spontaneous adverse drug reaction reports, gathered from a greater number of users, provide valuable information for all stakeholders in the healthcare system, from healthcare professionals to marketing authorization holders and regulatory authorities [4].

The importance of adverse drug reaction reporting has been recognized globally, leading to several initiatives aimed at improving reporting practices. For instance, national authorities in many countries (the United States, Canada, India, and a majority of European Union member countries), have developed and implemented mobile applications for adverse drug reaction reporting. Moreover, these mobile applications allow not only healthcare professionals but also consumers to report suspected adverse drug reactions. Previous research has shown that consumers’ active role in pharmacovigilance has proven useful [5,6]. Despite advancements in technology, health care professionals must proactively aim to ensure all individuals’ safety and health [7].

Pharmacists are increasingly recognized as integral members of the healthcare system and have the opportunity to expand their role in enhancing patient safety, especially the elderly and children. Building on this foundation, pharmacists have a unique opportunity to engage in pharmacotherapy monitoring and pharmacovigilance, particularly in managing adverse reactions and interactions between herbs or dietary supplements and medications [8,9,10]. As community pharmacists and their technicians are the most accessible health care professionals to patients, the aim of our study was to assess pharmacists’ and pharmacy technicians’ knowledge and practice of adverse drug reaction reporting. Furthermore, an additional aim was to analyze factors which could influence this practice.

## 2. Materials and Methods

A survey-based cross-sectional study was conducted in population of pharmacists and pharmacy technicians from Croatia in September 2022. The processed data was collected online via a Google Forms questionnaire, whose link was shared with pharmacy employees across Croatia with the help of a mentor and through mutual sharing. The study was previously approved by the Ethics Committee of the University of Split School of Medicine. The survey questionnaire used in this research was a translated and modified version of that developed by Li et al. [11]. The pilot study with 10 participants was conducted to assess content validity, readability, clarity, and comprehension of the survey questions. The validation process involved feedback collection through structured interviews and a thorough review by experts in the field to identify ambiguities or potential biases in the questions. Revisions were made based on this feedback to enhance clarity and ensure the survey’s reliability. Pharmacists and pharmacy technicians included in the validation process were excluded from the study to prevent any bias in the final results. To minimize response bias, particularly given that the questionnaire was self-administered, questions were carefully worded to avoid leading responses, and the survey was designed to be anonymous.

The questionnaire consists of three sections and contains a total of 28 questions and takes 5–10 min to complete. The first section consists of eight questions regarding sociodemographic data, such as gender, age, years of work experience, qualifications, place of degree attainment, field of work, use of drugs in chronic therapy, and the number of reported adverse drug reactions. The second section comprised six questions aimed at assessing knowledge about pharmacovigilance and reporting adverse reactions. It provided multiple-choice answers, with only one correct answer. It included knowledge of the definitions of pharmacovigilance and adverse reactions, examples of adverse drug reactions, the clinical trial phases for the same, types of adverse reactions, which suspected adverse reactions need to be reported and who can report them, and the most common safety reason for drug withdrawal from the market.

The third section of the questionnaire consists of a total of 14 questions, of which 12 questions use a five-point Likert scale (1—strongly disagree, 2—somewhat disagree, 3—neither agree nor disagree, 4—somewhat agree, 5—strongly agree). These questions assess the attitudes and practices of pharmacy employees regarding reporting all types of adverse reactions, the obligation for all healthcare professionals to report adverse reactions, the influence of incentives on the practice of reporting suspected adverse drug reactions, and the impact of the SARS-CoV-2 pandemic on raising awareness of the importance of reporting adverse reactions. The remaining two questions in the third section cover the frequency with which pharmacy employees encounter adverse drug reactions in patients and the most commonly used method for reporting suspected adverse reactions to the national authority, the Agency for Medicinal Products and Medical Devices. The questionnaire used in the study is available as Supplementary File S1.

Results are presented as whole numbers and proportions, mean ranks, and odds ratios. Kruskal–Wallis and Mann–Whitney tests were used to compare knowledge scores between participants based on their demographic characteristics, given that normality test showed abnormal data distribution. The Chi-square test and Fischer’s exact test were used to compare answers to Liker-scale questions and reporting adverse drug events between the groups of the participants. Variables proven significant by Chi-square test were chosen as predictors of the odds of reporting adverse drug events in multivariate logistic regression. Statistical analysis was performed using IBM SPSS Statistics software (version 25). Statistical significance was set at *p* < 0.05

## 3. Results

There were 180 participants in the study and the majority of them were female (85.56%). As seen in Table 1, most of the participants were less than 40 years old (72.78%). More than half of the participants had less than 10 years of work experience (57.78%), with only 16.67% of the participants having more than 20 years of work experience at the time of the study. The majority of the participants were pharmacists (79.44%), with the rest being pharmacy technicians. Participants mostly did not use drugs in a chronic manner (73.89%). More than a quarter of participants (27.22%) had never reported an adverse drug reaction, with more than a third (38.33%) of them reporting less than five adverse drug reactions in their careers.

A median total score in the adverse drug reactions knowledge section of the questionnaire was 4 out of 6 (IQR = 2). Table 2 shows the number and proportions of correct and incorrect answers on given questions in the previously mentioned section. Interestingly, the best answered question was about the type of adverse reactions that should be reported (96.11%), and the least amount of knowledge was shown on question requesting participants to recognize adverse reaction out of given ones (31.67%). Four out of six questions were predominantly answered correctly, and two questions predominantly incorrectly.

Different level of work experience had a significant impact on total score in the first section of the questionnaire as participants who had less than five years of work experience scored the highest mean rank (107.16) out of all other groups and the most experienced participants scored second lowest (74.90, *p* = 0.001). As seen in Table 3, pharmacists had significantly better knowledge when compared to pharmacy technicians (94.78 vs. 73.97, pharmacists’ and pharmacy technicians’ mean rank, *p* = 0.024), and participants who were chronic medication users also showed better understanding of the subject compared to those who do not use medication regularly (104.96 vs. 85.39, chronic medication users’ and non-users’ mean rank, *p* = 0.021). Additionally, participants’ knowledge about adverse drug reactions grows with the number of adverse drug reactions they reported. Participants who never reported adverse drug reaction had lower scores than participants who had reported 16–30 adverse drug reactions in their professional careers (81.07 vs. 126.70, 0 adverse drug reactions reported and 16–30 reported mean rank, *p* = 0.047), with mean rank values growing between the groups. Interestingly, the lowest score was accomplished by participants who reported the most adverse drug reactions (61.43, mean rank).

### 3.1. Attitudes on Reporting Adverse Drug Reactions

According to Table 4, most participants agree that reporting adverse drug reactions is a part of pharmaceutical care (97.23%) and that it should be mandatory for all pharmacy professionals (88.33%). Most of the pharmacists thought they had sufficient knowledge and training about reporting adverse drug reactions (70.78%) and stated that they are not afraid of legal consequences related to reporting adverse drug reactions (81.67%). Participants considered the reporting of adverse reactions to herbal medicines to be important (93,89%) but only a little more than half of them rated well-known adverse drug reaction as important (68.34%).

Answers to the third part of the questionnaire were compared between participants based on their demographic characteristics. There were no significant differences based on demographic characteristics between participants on questions in the Table 4, except that younger participants (18–30 years) would be more motivated to report adverse drug reactions (58.2%) while their older colleagues do not find reward to be sufficient motivation for reporting adverse drug reactions (*p* = 0.001, Fischer’s exact test).

### 3.2. Reporting Adverse Drug Reactions

The majority of the participants (72.78%) had reported adverse drug reactions in their professional careers. Table 5 illustrates that there is significant difference in the proportion of pharmacists that have reported adverse drug event compared to pharmacy technicians (80.40% vs. 43.20%, pharmacists and pharmacy technicians, *p* < 0.001). Further, participants who used medication on a daily basis reported adverse drug reactions in a higher proportion compared to those who did not (87.20 vs. 67.70, chronic medication users and non-users, *p* = 0.012).

### 3.3. ADR-Adverse Drug Reaction

Demographic characteristics that have proven significant for adverse drug reaction reporting proportions were chosen as independent variables in a logistic regression model to quantify their influence on adverse drug event reporting. A multivariate binary logistic regression model was significant in predicting the odds of reporting adverse drug event (Chi-square = 27.469 (df = 5), *p* < 0.001). As seen in Table 6, the odds of the participant reporting adverse drug reaction are 74.60% lower for participants who do not use medication chronically compared to those who do (*p* = 0.007), while pharmacists have 83.60% higher odds of reporting adverse drug reactions compared to pharmacy technicians (*p* <0.001). However, the Nagelkerke R-square is equal to 0.205, which means that selected predictors explain 20.50% of variability in the reporting of adverse drug reactions.

## 4. Discussion

The results of our study indicate that pharmacists are more likely to report adverse drug reactions compared to pharmacy technicians. Additionally, participants, regardless of their profession, were more inclined to report adverse drug reactions if they were also patients undergoing chronic pharmacotherapy. These findings suggest that personal experience, beyond formal education, significantly influences daily practice, particularly in pharmacovigilance activities. Similar results were observed in a previous study by our research group, which showed that personal experience with dietary supplement use also increased the likelihood of pharmacists recommending the same product to consumers [12]. Future studies should explore how personal experiences among all healthcare professionals influence their professional roles and potentially disrupt personalized patient care.

Interestingly, the results also revealed a higher level of pharmacovigilance knowledge among pharmacists compared to pharmacy technicians. Similar results were observed in studies by Al-Worafi et al. and Laven et al. [13,14]. Considering that pharmacy technicians in our research expressed dissatisfaction with their level of education on pharmacovigilance, it is possible that they are not adequately informed about how to report suspected adverse drug reaction to a competent authority. Furthermore, knowledge levels improved with the number of adverse drug reactions reported. These findings highlight a gap in integrating pharmacy technicians into pharmacovigilance activities, emphasizing the need to enhance their involvement to improve adverse drug reaction reporting and patient safety.

Pharmacy technicians are often involved in the self-medication process with patients, and their education should include training on how to recognize and report adverse drug reactions, as these could be the reason patients visit the pharmacy in the first place. The public should receive better education and be made aware of the consequences, risks, and potential adverse drug reactions of self-medication [15]. Awareness campaigns could play a significant role in helping patients evaluate the suitability of this practice. Pharmacists and pharmacy technicians have proven their ability to affect patient care through different programs, including medication optimization and educational consultations. Moreover, as shown by Valliant et al. [16], patients tend to visit their community pharmacies approximately one-and-a-half to two times as often as they visit their physicians or other health care professionals. The number of visits would have been even higher, given that the study included visits which were related to a prescription medicine only. Pharmacists play a major role in the health care system that is enhanced by their accessibility [17].

Although the results of our study showed high levels of knowledge among community pharmacists, even higher than knowledge levels observed in previous studies, not all of the study participants have reported adverse drug reaction to a regulator during their professional career [18]. Well-educated reporters such as pharmacists have an unquestionable role in the future improvement of the reporting system. In our research, it was observed that as many as 27.2% of respondents have never reported suspected adverse drug reactions. The issue of low activity among healthcare professionals in participating in pharmacovigilance activities has been recognized on a global scale. For newly approved drugs and those still under monitoring due to identified safety concerns, artificial intelligence tools are now being used worldwide to detect potentially life-threatening effects in this category of drugs [19]. Moreover, the completeness of adverse drug reaction reports is crucial for assessing putative causal relationships and, with the global average score of approximately 0.44 measured using the vigiGrade completeness score in VigiBase, the World Health Organization global database of reported potential adverse drug reactions shows huge potential for the improvement of the quality of reports [20].

In recent years, Croatia undertook several campaigns which aimed to raise awareness about the importance of pharmacovigilance among health care professionals, including Awareness Week on the Importance of Reporting Suspected Adverse Reactions. These campaigns have focused on the role of all healthcare professionals and patients in reporting suspected adverse reactions, thereby contributing to the safe use of medicines. Regulatory authorities and other stakeholders from numerous countries within the European Union and worldwide have been participating in the implementation of this global campaign for the last nine years, known as #MedSafetyWeek [21].

It is important to emphasize that awareness of the importance of pharmacovigilance has increased in the public and among healthcare professionals over the past few years, primarily due to the monitoring of the safety of newly approved COVID-19 vaccines. Although all healthcare professionals had the opportunity to participate in various projects related to vaccine safety monitoring, our results suggest that a portion of healthcare workers still has not integrated the reporting of suspected adverse drug or vaccine reactions into their routine practice in pharmacy settings. Given that one-third of respondents do not report suspected adverse drug reactions, it can be concluded that additional education and public health campaigns in the field of pharmacovigilance are needed [22]. Although our participants believed that the vaccines for the SARS-CoV-2 pandemic have increased awareness about the importance of reporting adverse effects, real data show that there was no effect on the number of reported events related to the medicines. As can be seen in the national authority annual report, the number of reported events in the 2021, a year when SARS-CoV-2 vaccines became available for the population, increased more than 100% (9996 reported events); however, this number increased due to reported events related to vaccines only, while the number of reported events related to other medicines remained stable [23].

Our results indicate that pharmacists with less working experience demonstrated higher levels of pharmacovigilance knowledge. It is important to highlight that, in addition to the general obligation of all healthcare professionals to report adverse drug reactions to the national authority, the Croatian Pharmacy Competency Framework explicitly emphasizes this responsibility. Within the “Pharmaceutical Care” cluster, Competency 2.5, “Ensuring the Safe Use of Medicines,” specifically outlines activity 2.5.5: identify and report suspected adverse drug reactions to the relevant authority. Thus, ADR reporting should be regarded as standard practice for all pharmacists. The competency framework encompasses core competencies for pharmacists with up to five years of independent professional experience. This responsibility is particularly well-integrated among pharmacy graduates from the past decade, whose professional training during the fifth year of study and final exams is structured around mastering the competencies defined in this framework [24]. Future studies should aim to propose strategies for enhancing continuing education in pharmacovigilance for practicing pharmacists and pharmacy technicians. These efforts would further strengthen the implementation of adverse drug reaction reporting as an integral part of pharmaceutical care.

An interesting example of pharmaceutical care improvement initiatives is described in a study by Spanakis et al., where the authors discuss the implementation of the PharmActa mobile application in Greece. The application enables pharmacists to review patients’ medical histories and empowers patients to become more knowledgeable about medication safety [25]. Furthermore, recently published data on adverse drug reaction underreporting identified a lack of time as the main barrier to reporting. Future initiatives should address this issue [26]. The current evidence regarding which interventions (e.g., informational emails or financial incentives) would effectively improve underreporting remains uncertain [27].

Certain limitations exist in this study. The first limitation to emphasize is the small number of respondents, especially pharmacy technicians. Although their proportion in this study is small, this is among the first studies to include pharmacy technicians in addressing the issue of pharmacovigilance. According to the draft plan of the new Pharmacy Act, pharmacy technicians in the Republic of Croatia will also have authorization for independent practice. To renew this authorization, they will need to collect points within a specific period by participating in educational activities or reporting suspected adverse drug reactions. Thus, it can be expected that, in the future, the number of reports from pharmacy technicians will increase as their knowledge on this topic improves. The second limitation is the survey questionnaire, which was completed via personal computers or mobile devices, raising the possibility that respondents may have checked answers while completing the survey. Despite this, it can be considered that conducting this study has raised awareness among pharmacists and technicians about pharmacovigilance and the existence of a mobile application, which could result in an increased number of adverse drug reaction reports from them in the future.

## Figures and Tables

**Table 1:** Demographic characteristics of participants.

		N	Proportion
Sex	Female	154	85.56%
	Male	26	14.44%
Age	18–30	67	37.22%
	31–40	64	35.56%
	41–50	37	20.56%
	51–60	9	5.00%
	61–70	3	1.67%
Work experience in years	<5	65	36.11%
	5–10	39	21.67%
	10–15	22	12.22%
	15–20	24	13.33%
	>20	30	16.67%
Profession	Pharmacy technician	37	20.56%
	Pharmacist	143	79.44%
Chronic use of drugs	No	133	73.89%
	Yes	47	26.11%
Number of reported adverse drug reactions	0	49	27.22%
	<5	69	38.33%
	5–15	45	25.00%
	16–30	10	5.56%
	>30	7	3.89%

**Table 2:** Participants’ knowledge of pharmacovigilance activities.

		Count	N%
Pharmacovigilance definiton	Incorrect	25	13.89%
	Correct	155	86.11%
Adverse drug reaction example	Incorrect	123	68.33%
	Correct	57	31.67%
Type of reaction national authority requires healthcare professionals to report	Incorrect	7	3.89%
	Correct	173	96.11%
Acceptable reporters of adverse drug reactions	Incorrect	11	6.11%
	Correct	169	93.89%
Phase where the most data on drug safety are collected	Incorrect	65	36.11%
	Correct	115	63.89%
Most common safety reason for withdrawal of a drug from the market	Incorrect	116	64.44%
	Correct	64	35.56%

**Table 3:** Comparison of total knowledge score of participants based on demographic characteristics.

		Mean Rank	*p*-Value
Sex	Female	91.41	0.553
	Male	85.12	
Age	18–30	91.03	0.647
	31–40	96.61	
	41–50	82.19	
	51–60	79.00	
	61–70	85.33	
Work experience in years	<5	107.16	0.001 *
	5–10	68.15	
	10–15	104.20	
	15–20	88.63	
	>20	74.90	
Profession	Pharmacy technician	73.97	0.024 *
	Pharmacist	94.78	
Chronic use of medication	No	85.39	0.021 *
	Yes	104.96	
Number of reported adverse drug reactions	0	81.07	0.047 *
	<5	92.97	
	5–15	93.46	
	16–30	126.70	
	>30	61.43	

* *p* < 0.05, Mann–Whitney test for two category variables; Kruskal–Wallis test for multiple category variables.

**Table 4:** Likert-scale answers on the third part of the questionnaire regarding participants’ attitudes on reporting adverse drug reactions.

	Strongly Disagree	Disagree	Nor Agree or Disagree	Agree	Strongly Agree
	Count (%)	Count (%)	Count (%)	Count (%)	Count (%)
Reporting adverse effects is a part of pharmaceutical care.	0 (0.00)	1 (0.56)	4 (2.22)	75 (41.67)	100 (55.56)
Reporting adverse effects should be mandatory for all pharmacy professionals.	3 (1.67)	3 (1.67)	15 (8.33)	81 (45.00)	78 (43.33)
I do not have time to report adverse effects during my work hours.	30 (16.67)	45 (25.00)	53 (29.44)	38 (21.11)	14 (7.78)
Patients do not share information about adverse effects with us.	15 (8.33)	57 (31.67)	66 (36.67)	35 (19.44)	7 (3.89)
I believe that the vaccines for the SARS-CoV-2 pandemic have increased awareness about the importance of reporting adverse effects.	4 (2.22)	26 (14.44)	68 (37.78)	69 (38.33)	13 (7.22)
I currently have sufficient knowledge and training on how to report adverse effects.	1 (0.56)	20 (11.11)	28 (15.56)	91 (50.56)	40 (22.22)
I am concerned that reporting adverse effects to the national authority may lead to legal consequences.	63 (35.00)	84 (46.67)	24 (13.33)	8 (4.44)	1 (0.56)
I believe it is not necessary to report adverse effects to herbal medicines.	88 (48.89)	81 (45.00)	8 (4.44)	2 (1.11)	1 (0.56)
I would be motivated to report adverse effects if I were rewarded for doing so.	20 (11.11)	29 (16.11)	57 (31.67)	44 (24.44)	30 (16.67)
I believe there is no need to report well-known adverse effects.	55 (30.56)	68 (37.78)	31 (17.22)	22 (12,22)	4 (2.22)

**Table 5:** Comparison of participants who had reported adverse drug reactions based on demographic characteristics.

		Proportion of Participants That Had Report ADR	*p*-Value
Age	18–30	64.2	0.160
	31–40	71.9	
	41–50	83.8	
	51–60	88.9	
	61–70	100.0	
Work experience in years	<5	64.6	0.326
	5–10	74.4	
	10–15	81.8	
	15–20	70.8	
	>20	83.3	
Profession	Pharmacy technician	43.2	<0.001 *
	Pharmacist	80.4	
Chronic use of medication	No	67.7	0.012 *
	Yes	87.2	

* *p* < 0.05, Chi-square test or Fischer’s exact test.

**Table 6:** Multivariate binary logistic regression results for factors associated with reporting of adverse drug reactions.

Variable	B	Exp (B)	95%CI for B		*p*-Value
			Lower Bound	Upper Bound	
Chronic use of medication					
Yes	Ref.				
No	−1.369	0.254	0.094	−0.256	0.007 *
Profession					
Pharmacist	ref.				
Pharmacy technician	−1.810	0.164	−2.430	0.073	<0.001 *

* *p* < 0.05, logistic regression.

## Data Availability

The raw data supporting the conclusions of this article will be made available by the authors upon request.

## Supplementary Materials

The following supporting information can be downloaded at: https://www.mdpi.com/article/10.3390/pharmacy13010001/s1, File S1: ADR Knowledge and Practice among Pharmacy Professionals Survey Questionnaire.
