# Supplementary material for: Empowering Patient Safety: Assessment of Adverse Drug Reaction Knowledge and Practice Among Pharmacy Professionals
Source: Pharmacy (Basel). 2024 Dec 29;13(1):1. doi: 10.3390/pharmacy13010001 (PMC11755484; doi:10.3390/pharmacy13010001)
Supplement: Supplementary file 1 [file pharmacy-13-00001-s001.zip › pharmacy-3361759-supplementary.pdf]

What is the definition of pharmacovigilance?

a) Detection, assessment, understanding, prevention of adverse effects* b) Reporting adverse effects c) The science of improving the safety of medical products d) The science of evaluating the benefits and risks of medical products e) I don’t know

What is an example of an adverse event?

a) A patient taking drug X is hospitalized due to a stroke. The hospital doctor believes that drug X did not cause the stroke. b) A patient taking drug X developed a rash. It is believed that the rash was caused by drug X. c) A patient taking drug X said that the drug X is not effective. They did not experience any adverse effects. d) All of the above* e) I don’t know

What type of adverse effects does HALMED request healthcare professionals to report in Croatia?

a) Serious and unexpected adverse effects b) Non-serious adverse effects c) Expected adverse effects d) All adverse effects regardless of seriousness and expectation* e) I don’t know

In Croatia, which organizational unit of HALMED is responsible for collecting and monitoring adverse effects?

a) Department for Drug Approval b) Department for Legal, Economic, IT, and General Affairs c) Department for the Safe Use of Medicines and Medical Devices* d) Official Medicines Control Laboratory (OMCL) e) I don’t know

Who can report adverse effects of medicines to HALMED?

a) Only consumers and patients b) Only healthcare professionals c) Only hospitals d) All of the above* e) I don’t know

How does TGA communicate important or new ADR (Adverse Drug Reaction) information that has been confirmed?

a) TGA Early Warning System – communication monitoring b) TGA Drug Safety Bulletins* c) TGA Risk Management Plan d) All of the above e) I don’t know

At which stage is the most data on drug adverse events collected?

a) Phase I clinical trials b) Phase II clinical trials c) Phase III clinical trials d) Phase IV clinical trials and post-marketing surveillance* e) I don’t know

Consider the following 3 statements. Choose the correct answer:

I. The terms “adverse event” and “adverse reaction” are synonyms and can be used interchangeably. II. An adverse event is considered anything medically undesirable, and there is no need to establish a causal relationship between the adverse event and the drug. III. Adverse reactions are collected during clinical trials, while adverse events are collected during the post-marketing period.

a) Only statement I is correct b) Only statement II is correct* c) Only statements I and II are correct d) Only statements II and III are correct e) I don’t know

Which of the following events would HALMED not always consider a serious adverse event?

a) An adverse event resulting in hospitalization b) A severe adverse event* c) An adverse event resulting in a congenital anomaly/birth defect d) An adverse event considered serious by a healthcare professional e) I don’t know

What is the most common safety reason for withdrawing a drug from the market?

a) Hepatotoxicity* b) Cardiotoxicity c) Carcinogenicity d) Nephrotoxicity e) I don’t know

Reporting adverse reactions is important for patient well-being. Strongly disagree - Disagree - Neutral - Agree - Strongly agree

Reporting adverse reactions should be mandatory for all pharmaceutical professionals. Strongly disagree - Disagree - Neutral - Agree - Strongly agree

I do not have time to report adverse reactions during my work hours. Strongly disagree - Disagree - Neutral - Agree - Strongly agree

It is necessary to teach pharmacovigilance in university pharmacy programs. Strongly disagree - Disagree - Neutral - Agree - Strongly agree

Professional/official bodies should organize workshops or training to raise awareness of the importance of reporting adverse reactions. Strongly disagree - Disagree - Neutral - Agree - Strongly agree

I currently have sufficient knowledge and training on how to report adverse reactions. Strongly disagree - Disagree - Neutral - Agree - Strongly agree

I am afraid of potential legal consequences if I report adverse reactions to HALMED. Strongly disagree - Disagree - Neutral - Agree - Strongly agree

I have a professional obligation to report adverse reactions. Strongly disagree - Disagree - Neutral - Agree - Strongly agree

There are no changes or consequences from the adverse reactions I have reported. Strongly disagree - Disagree - Neutral - Agree - Strongly agree

I would be motivated to report adverse reactions if I were rewarded for doing so. Strongly disagree - Disagree - Neutral - Agree - Strongly agree

In my pharmacy dispensing software system, there is a reminder. Strongly disagree - Disagree - Neutral - Agree - Strongly agree

Patient data is automatically filled in from the pharmacy dispensing software into the adverse reaction reporting form. Strongly disagree - Disagree - Neutral - Agree - Strongly agree

General education on the importance of pharmacovigilance is ensured. Strongly disagree - Disagree - Neutral - Agree - Strongly agree

How often do you observe adverse reactions in patients?

a) At least once a week b) At least once a month c) At least once a year d) Never

How often do you document adverse reactions as part of a clinical intervention?

a) At least once a week b) At least once a month c) At least once a year d) Never

What is the most common method you use to report suspected adverse drug reactions to HALMED?

a) Online reporting b) Mobile application c) OPeN system for healthcare professionals d) Adverse reaction reporting form e) Telephone, fax f) I have not reported any adverse reactions to HALMED

Finally, do you have any suggestions that would encourage you to report more suspected adverse drug reactions?
